# The Role of Intestinal Microbiota in Colorectal Cancer

**DOI:** 10.3389/fphar.2021.674807

**Published:** 2021-04-19

**Authors:** Lingli Ren, Juan Ye, Bing Zhao, Jinbing Sun, Peng Cao, Yang Yang

**Affiliations:** ^1^ Affiliated Hospital of Integrated Traditional Chinese and Western Medicine, Nanjing University of Chinese Medicine, Nanjing, China; ^2^ Department of Pharmacology, School of Pharmacy, Nanjing University of Chinese Medicine, Nanjing, China; ^3^ Department of General Surgery, Changshu No. 1 People’s Hospital, Affiliated Changshu Hospital of Soochow University, Changshu, China; ^4^ Jiangsu Key Laboratory for Pharmacology and Safety Evaluation of Chinese Materia Medica, Nanjing University of Chinese Medicine, Nanjing, China; ^5^ Yangtze River Pharmaceutical Group, Taizhou, China

**Keywords:** colorectal cancer, intestinal flora, immune-inflammation, metabolism, prevention and treatment of colorectal cancer

## Abstract

Colorectal cancer is a multifactorial disease involving genetic, environmental, and lifestyle risk factors. Intestinal microbiota plays an important role in the occurrence and development of colorectal cancer. Studies have shown that the behavior of intestinal microbiota can lead to pathological changes in the host intestine, which can be divided into epigenetic changes and carcinogenic changes at the gene level, and ultimately promote the formation and development of colorectal cancer. Intestinal microbiota is mainly distributed in the intestinal epithelium, which is composed of a large number of microorganisms interacting with the host intestinal cells. It can affect the immune-inflammation and metabolism of the gastrointestinal tract, and may be used as a biomarker for disease diagnosis. Regulation of gut microbiota is a promising strategy for the prevention and treatment of colorectal cancer. This article reviews the role of intestinal microbiota in the development of colorectal cancer, including the related mechanisms of intestinal microbiota promoting colorectal cancer, the use of intestinal microbiota in the diagnosis of colorectal cancer, and the regulation of intestinal microbiota in the prevention or treatment of colorectal cancer.

## Introduction

Colorectal cancer (CRC) accounts for about 10% of the new cancer cases worldwide. Its incidence rate is third among all cancers worldwide, and its mortality rate ranks second among all cancers (Bray et al., 2018). The formation of CRC involves complex changes of multiple genes, steps, and stages, and various genetic and environmental factors are related to the occurrence and development of CRC (Theodoratou et al., 2017). Recently, more and more attention has been paid to the role of microorganisms in cancer. Researchers have begun to study the impact of changes in microbial communities on cancer. These microbial communities have become an important influencing factor of some cancers, including CRC, liver cancer, breast cancer, and so on. The human intestinal tract is one of the most complex organs of the human body, which hosts tens of thousands of microorganisms, including bacteria, archaea, fungi, protozoa, and viruses, of which bacteria account for the majority (Gill et al., 2006). Intestinal microflora mainly act on intestinal epithelial cells, interact with intestinal cells, maintain intestinal environment, and play an important role in human health, such as energy metabolism (Turnbaugh et al., 2006) and immune regulation (Chung et al., 2012). The change in its relative abundance will disrupt the balance of the intestinal microenvironment, thus causing some diseases inside and outside the intestine. This review provides an overview of the relationship between intestinal microbiota and CRC, focusing on the mechanism of intestinal microbiota in CRC, and finally discusses the potential strategies for the prevention or treatment of CRC based on the regulation of intestinal microbiota.

## Intestinal Microbiota and Colorectal Cancer

**FIGURE 1:**
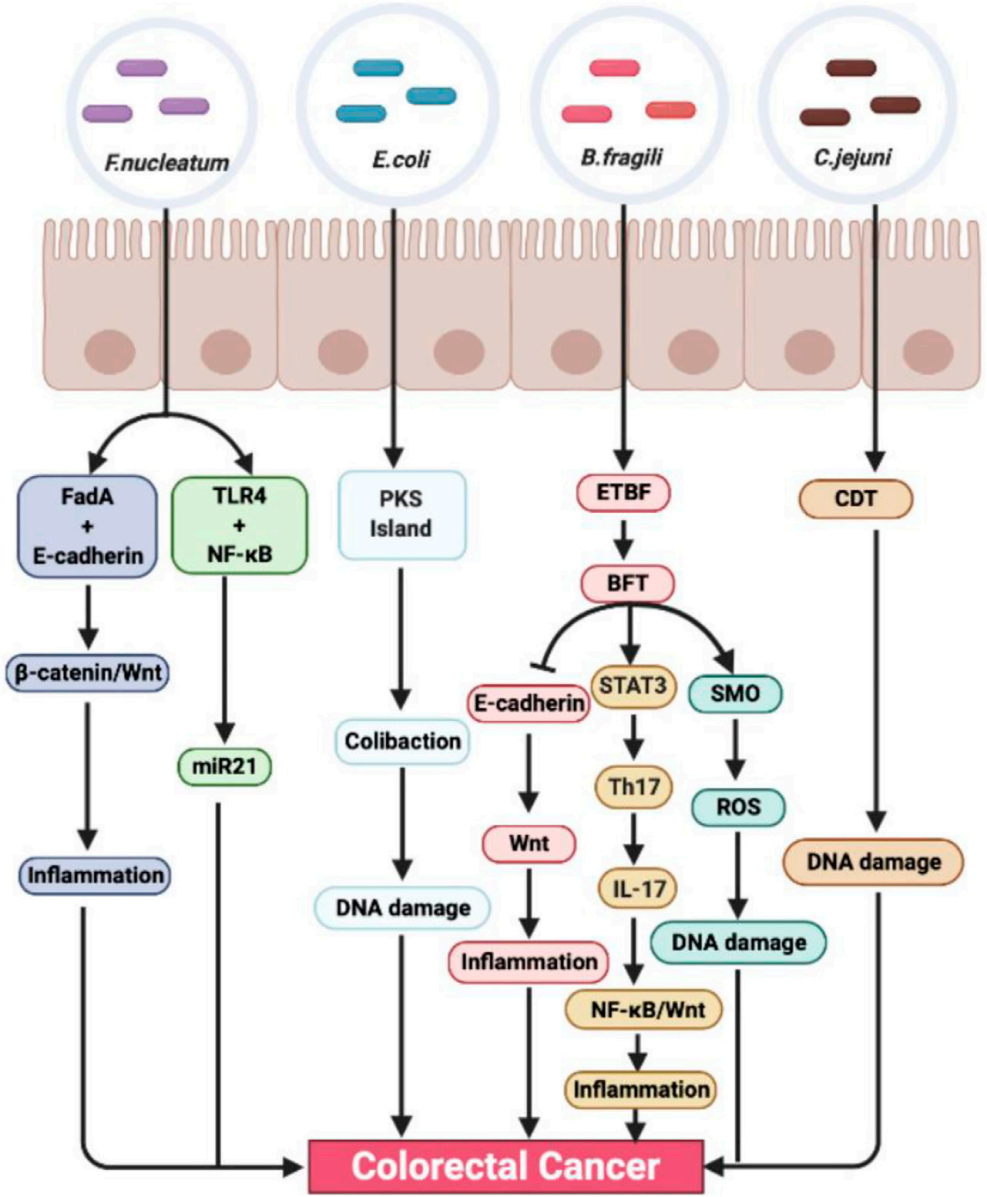
The role and mechanism of intestinal microbiota promoting colorectal cancer. *Fusobacterium nucleatum, E. coli, B. fragili, and C. jejuni* are closely related to the occurrence and development of colorectal cancer. It can stimulate inflammation and promote tumor formation through many inflammation-related signaling pathways, such as NF-κB, STAT3, and Wnt/β-catenin. At the same time, intestinal flora can also cause DNA damage, lead to gene mutation, and promote tumor formation through its own toxins.

There are hundreds of microorganisms in the intestine, which form a symbiotic system with intestinal cells to maintain a dynamic balance and maintain the intestinal environment. Once the balance is destroyed, the intestinal flora will be out of balance, causing a series of intestinal diseases. As early as the 1960s, the relationship between intestinal flora and CRC was studied. In this study, through the induction of conventional rats and sterile rats, it can be found that *Cycas* has carcinogenic effect on conventional rats, but failed to cause cancer in sterile rats, which indicates that intestinal microorganisms play an important role in the carcinogenesis mediated by *Cycas* (Laqueur et al., 1967). Recently, through a case-control study of CRC patients, a research team analyzed the characteristics of fecal flora and blood inflammatory factors in various stages of colorectal tumorigenesis (benign polyps to advanced adenoma), identified 24 CRC-related bacteria, and revealed that the flora is involved in promoting the formation of CRC microenvironment in the process of gradual malignant transformation (Zhang et al., 2018). At present, the known flora associated with CRC mainly includes *Fusobacterium nucleatum* (*F. nucleatum*), *Escherichia coli* (*E. coli*), *Bacteroides fragilis* (*B. fragilis*), *Campylobacter jejuni* (*C. jejuni*), etc. (Dai et al., 2019) (Figure 1).

### Fusobacterium nucleatum

*Fusobacterium nucleatum* is a common symbiotic anaerobic Gram-negative bacteria in oral cavity. It is unique to human oral cavity and plays an important role in periodontal disease. It can bridge different microorganisms in biofilm through a variety of adhesins. More and more evidence show that *F. nucleatum* affects many stages of CRC development. *Fusobacterium nucleatum* can increase the proliferation of cancer cells through two different mechanisms: 1) The binding of FadA and E-cadherin drives the activation of *β*-catenin and Wnt pathway (Rubinstein et al., 2013). 2) The activation of TLR4 and NF-κB leads to the increased expression of carcinogenic miR21 (Yang et al., 2017). The researchers verified the above conclusion by constructing an intestinal tumor model in Apc^min/+^ mice, and found that compared with the control group, *F. nucleatum* can cause Apc^min/+^ mice to exhibit high levels of proliferating cell nuclear antigen, promote cell proliferation, and increase the level of inflammatory mediators in mouse serum and myeloid cells infiltrating into the tumor, thus promoting the development of tumor (Kostic et al., 2013). In addition, *F. nucleatum* may also affect the metastatic transmission. It can work together with other species and genera of bacteria such as *Bacteroides*, *Selenomonas,* and *Prevotella* to promote the metastasis of CRC, and can be isolated from liver and lymph node metastasis (Bullman et al., 2017). After CRC treatment, *F. nucleatum* can inhibit mir-18a* and mir-4802 involved in autophagy, thus increasing the risk of CRC recurrence and chemotherapy resistance (Yu et al., 2017). In addition, *F. nucleatum* also promotes the glycolysis and carcinogenesis of CRC by upregulating the histone modification of ENO1 (a key component in the glycolysis pathway) and other genes by upregulating the long non-coding RNA enolase 1 (ENO1)-intron transcript (ENO1-IT1)] (Hong et al., 2020).

### Escherichia coli

*Escherichia coli* is a kind of anaerobic Gram-negative symbiotic bacteria, which colonizes the human intestinal tract shortly after birth. However, some virulent strains of *E. coli* with pathogenicity island can infect human gastrointestinal system and induce diseases. *E. coli* strains are divided into four main phylogenetic groups: A, B1, B2, and D. Fecal strains often belong to groups A and B1, while the most common pathogenic strains carrying virulence factors belong to groups B2 and D (Smati et al., 2013). Some pathogenic strains in groups B2 and D are associated with inflammatory bowel disease (IBD), and IBD is a risk factor for CRC. Colibactin is a bacterial gene toxin first mentioned by Nougayrede et al., in 2006. It is mainly produced by *E. coli*. Some studies have shown that colibactin is closely related to CRC, which can cause double-strand DNA breakage, eukaryotic cell cycle arrest, and chromosome aberration, and induce CRC. In the CRC mouse model, colibactin-producing *E. coli* can promote the occurrence of colon cancer (Arthur et al., 2012). In human CRC cells, colibactin preferentially destroys DNA rich in AT-enriched hexamer sequence motifs, which is related to different DNA shape characteristics and electrostatic potential. Somatic mutations of colibactin targets were analyzed in thousands of cancer genomes, and it was found that colibactin-binding sequences aggregated in CRC (Dziubanska-Kusibab et al., 2020).

### Bacteroides fragilis

*Bacteroides fragilis* is a kind of common bacteria in the intestinal tract, which is symbiotic with the host. Most bacteria help the human body digest food and maintain intestinal health. However, in some cases, these bacteria produce a toxin that disrupts the cells on the surface of the gut, thereby promoting CRC. There are two subtypes of *B. fragilis*, namely, nontoxic *B. fragilis* and enterotoxigenic *B. fragilis* (ETBF), which produce a kind of *B. fragilis* toxin (BFT). BFT can lead to the destruction of epithelial barrier and the cleavage of E-cadherin. At the same time, the cleavage of E-cadherin can activate the Wnt signal transduction pathway, stimulate mucosal inflammation, and promote the formation of colon tumor. In addition, the STAT3 pathway is the target of colon transformation, and it is also necessary for the development of Th17 cells. ETBF promotes the production of IL-17 b y Th17 cells through the rapid activation of the STAT3 pathway, which further promotes the activation of the NF-κB and Wnt pathways and the production of intestinal inflammatory tumor microenvironment. This process depends on the production of IL-17, and injection of IL-17 blocking antibody in mice can inhibit tumor formation (Dejea et al., 2013). ETBF can also upregulate the expression of spermine oxidase (SMO) in colonic epithelial cells, thus increasing the SMO-dependent reactive oxygen species (ROS), promoting the release of inflammatory cytokines and causing DNA damage, and ultimately promoting the development of CRC.

### Campylobacter jejuni

As early as 20 years previously, it was reported that some *Campylobacter*, including *C. jejuni*, had cytolethal distending toxin (CDT) (Lara-Tejero and Galan, 2000). CDT has dnase activity, leading to DNA double-strand breaks. Zheng He et al. (Yusuf et al., 2019) found that *C. jejuni* 81–176 isolated from human body was colonized in sterile Apc^min/+^ mice, which could significantly increase the number and size of tumor, and the macro transcriptome and intestinal flora of mice were also significantly changed. *C. jejuni* has a mutated CDTb subunit, which makes the bacteria unable to produce CDT, thus inhibiting its carcinogenesis *in vivo* and reducing the DNA damage response of cells and intestinal organs.

## Mechanism of Intestinal Microbiota Promoting Colorectal Cancer

The changes in intestinal microbiota abundance and intestinal microecological structure will lead to an imbalance of intestinal microbiota. Once the intestinal microbiota is unbalanced, the number of probiotics in the intestine will decrease, and the number of pathogenic bacteria will increase. Pathogenic bacteria will secrete a variety of toxic factors, damage intestinal epithelial cells, and cause chronic inflammatory reaction. Meanwhile, in the process of inflammation, a variety of cytokines and chemokines will be released to activate inflammation-related signaling pathways, thus promoting CRC. In addition, epigenetic modification enables host cells to alter gene expression and sequence. Microorganisms can interact with host genomes dynamically through the interface of epigenetic modification. Epigenetics also plays an important role in the occurrence and development of CRC.

### Intestinal Microbiota and Immune Response

Intestinal microbiota promotes the maturation and regulation of mucosal and systemic immune systems through innate and adaptive immune cells. For example, the activation of intestinal cells, macrophages, pattern recognition receptors, and highly specific receptors on the surface of T cells and B cells in the mucosa is highly regulated by intestinal microorganisms (Lucas et al., 2017). Studies have shown that intestinal microflora can activate mucosal macrophages through intestinal mucosal M cells and trigger innate immune response, thus promoting the occurrence and development of CRC (Grivennikov et al., 2012). In adaptive immune response, including humoral response, soluble tumor antigen is recognized by specific B cells, matures, and produces tumor antigen-specific antibody with the help of CD4^+^ T cells (Vesely et al., 2011). Adaptive immunity plays an important role in regulating the composition of intestinal microflora, and the intestinal microbiota of mice lacking adaptive immunity will change accordingly (Kato et al., 2014; Zhang et al., 2015). Helper T cells, T cells, and B cells secrete immunoglobulin A (IgA) and participate in tumorigenesis through adaptive immune response (Palm et al., 2015). The main mechanism of T cells affecting the composition of intestinal microorganisms is to assist B cells to produce IgA in the intestine, and IgA is mainly secreted into the intestinal cavity, which can bind and cover the intestinal microorganisms and participate in shaping the intestinal microbiota.

### Intestinal Microbiota and Immune Signal Pathway

IBD is an inflammatory bowel disease, including ulcerative colitis and Crohn's disease. CRC is closely related to IBD. Intestinal microbiota can induce mucosal lesions through pro-inflammatory cytokines, thus promoting IBD and increasing the risk of CRC (Ullman and Itzkowitz, 2011; Manichanh et al., 2012). Inflammatory cells release cytokines, such as IL-6, IL-8, IL-17, and TNF-α, which play an important role in the development of CRC. Studies have shown that *Enterococcus faecalis* (*E. faecalis*) infection can cause colitis and express TGF-β in intestinal epithelial cells, thus activating the Smad4 signaling pathway (Szigeti et al., 2012). *E. faecalis* can also produce ROS and H_2_O_2_, leading to DNA damage, thus activating NF-κB signaling pathway, stimulating inflammation, and affecting the occurrence and development of CRC. At the same time, it seems to be involved in the bystander effect of COX-2, that is, macrophages release TNF-α, causing chromosome instability and cell transformation, leading to CRC (Wang et al., 2013). In addition, *Helicobacter pylori* (*H. pylori*) can cause colorectal epithelial damage through IL-8-mediated inflammatory response, thus promoting the occurrence and development of CRC (Rubin et al., 2012). IL-17 is an important cytokine produced by Th17 cells, and the activation of STAT3 is required for Th17 cell growth. Two transcription factors, NF-κB and STAT3, are necessary for inflammation to promote the occurrence and development of cancer. ETBF can rapidly activate the STAT3 signaling pathway and promote Th17 cells to secrete IL-17; thus, IL-17-dependent NF-κB and Wnt pathways are activated to establish inflammatory tumor microenvironment in the gut (Goktuna et al., 2016; Housseau et al., 2016).

### Intestinal Microbiota and Epigenetic Modification

Epigenetic modification is heritable and potentially reversible. It is the central mechanism of transcriptional response to environmental cues and regulates gene expression through DNA modification, histone modification, and non-coding RNA. Studies have shown that intestinal flora can directly or indirectly regulate epigenetic modification and affect the occurrence and development of CRC. Epigenetic mechanisms that play an important role in the occurrence and development of CRC include DNA methylation, histone modification, miRNA and non-coding RNA, and nucleosome localization (Sharma et al., 2010). miRNA-21 and miRNA-200 b are two carcinogenic miRNAs, which are often upregulated in colorectal cancer cells. However, the expression levels of miRNA-21 and miRNA-200 b were significantly decreased in HT-29 cells treated with *Leuconostoc mesenteroides* (Zununi Vahed et al., 2017). In addition, intestinal microbiota metabolites can regulate cell differentiation through epigenetics, such as T cell development. Symbiotic bacteria, such as *Clostridium*, can promote the production of colon ptreg cells through its fermentation product butyric acid, which is an HDAC inhibitor and can promote the acetylation of Foxp3 promoter and histone H3 in CNS1 (Furusawa et al., 2013). At the same time, acetic acid and propionic acid promote the accumulation of colon Treg cells by activating GPR43 (Smith et al., 2013). Both induce Foxp3^+^ and CD4^+^ Treg cells, which play a key role in limiting the inflammatory response during carcinogenesis. In addition, butyric acid can increase the histone methylation of NF-κB1 promoter, thereby downregulating the expression of NF-κB1. Short-chain fatty acids (SCFAs) induce the expression of anti-inflammatory IL-10 R A (IL-10 receptor alpha subunit mRNA) and antimicrobial peptides through HDAC inhibitor function. Therefore, with the downregulation of NF-κB1, SCFAs can inhibit colitis through epigenetic modification. In addition, the fecal bacteria transplantation experiment found that the intestinal flora can also affect the expression of tumor-related genes by increasing DNA methylation of multiple CpG sites in CRC cells (Yu et al., 2015).

## Intestinal Microbiota and Diagnosis of Colorectal Cancer

Intestinal flora plays an important role in the diagnosis of CRC. It can be used as a biomarker in the diagnosis and prediction of CRC. Some studies revealed the characteristics of intestinal microflora that can predict CRC through metagenomic analysis of feces **(** Table 1 **)**. In this study, through metagenomic analysis of feces of people from different countries, different living habits and eating habits, bacteria associated with CRC were identified, such as *Fusobacterium*, *Porphyromonas*, *Parvimonas*, etc., which can be used as biomarkers for the diagnosis of CRC. In addition, they have also obtained two important research results: 1) The discovery of a special bacterium in CRC patients, *F. nucleatum*, which is common in the oral cavity and respiratory tract. Compared with healthy individuals, the level of *F. nucleatum* in CRC patients is higher. 2) The discovery of the correlation between CRC and microbial enzyme genes, which is an important microorganism in the feces of CRC patients Choline trimethylamine lyase (cutC) gene is a biological enzyme, which can degrade choline in meat and release acetaldehyde (Wirbel et al., 2019). This finding was also confirmed by Thomas et al. (Thomas et al., 2019), which indicates that intestinal bacteria in feces may be able to predict CRC. In addition, the assessment of gene changes in stool samples can accurately reflect the status of intestinal microbiota, which may provide important clues for the diagnosis and cause and effect of diseases and contribute to the early diagnosis of cancer (Yachida et al., 2019).

**TABLE 1:** Summary of harmful and beneficial bacteria in colorectal cancer.

	Phyla	Genus/Species	Abundance changes	Mechanism	References
Harmful bacteria	Proteobacteria	*Helicobacter*	↑	Inflammatory	Kamada et al. (2013)
		*Escherichia coli*	↑	Colibactin and DNA damage	Dziubanska-Kusibab et al. (2020)
	Bacteroidetes	*Bacteroides fragilis*	↑	BFT toxin	Chung et al. (2018)
				Activation of STAT3	
				Th-17 immune response induction	
				Production of IL-1	
				Activation of *β*-catenin signaling	
		*Bacteroides vulgatus*	↑	NF-κb activation	Karrasch et al. (2007)
		*Prevotella spp*	↑	Inflammatory	Dai et al. (2018)
		*Alistipes*	↑	Inflammatory	
	Fusobacteria	*Fusobacterium nucleatum*	↑	FadA binding to E-cadherin	Allen-Vercoe and Jobin (2014)
				Activation of *β*-catenin signaling	
				NF-κb activation Production of IL-6, IL-8 Myc, cyclin D1 activation	
	Firmicutes	*Streptococcus bovis*	↑	Expression of COX-2	Dai et al. (2019)
		*Parvimonas*	↑	Inflammatory, immune response	Allali et al. (2015)
		*Clostridium* spp.	↑	ROS production and DNA damage	Flemer et al. (2018)
				Production of DCA	
		*Enterococcus faecalis*	↑	ROS production and DNA damage	Dai et al. (2019)
		*Peptostreptococcus*	↑	Oxidative stress	Tsoi et al. (2017)
	Actinobacteria	*Atopobium parvulum*	↑	The central hub of H2S producers	Mottawea et al. (2016)
		*Slackia*	↑	Anti-oxidant potential	Marchesi et al. (2011)
Beneficial bacteria	Proteobacteria	*Shigella* spp.	↓	Shiga and shiga-like toxins	Koliarakis et al. (2018)
		*Salmonella* spp.	↓	Shiga and shiga-like toxins	
	Firmicutes	*Lactobacillus* spp.	↓	Immune modulatory (activation T-cells)	Chen et al. (2012)
				Mucus barrier maintenance	
		*Roseburia* spp.	↓	Anti-inflammatory	Shen et al. (2020)
				Butyrate production	
	Actinobacteria	*Bifidobacterium* spp.	↓	Immune modulatory	Feng et al. (2015)
				Anti-Inflammatory	
				Butyrate production	

## Intestinal Microbiota and Prevention of Colorectal Cancer

### Diet

CRC is a multifactorial disease, and changes in diet and lifestyle can aggravate the incidence and mortality rates of CRC. Excessive intake of animal protein and fat produces excessive secondary bile acids and hydrogen sulfide, which leads to barrier dysfunction, inflammation, DNA damage, genotoxicity, etc., and increases the risk of CRC, whereas dietary fiber produces SCFAs, such as butyrate, which plays anti-inflammatory and anti-tumor roles through cell metabolism, bacterial homeostasis, anti-proliferation, immune regulation, and epigenetics (Olagnier et al., 2018). In a follow-up study of more than 170,000 people with an average of 5.7 years, it was found that eating more red meat and drinking alcohol may increase the risk of CRC, while eating more fiber in breakfast may reduce the risk of CRC (Bradbury et al., 2020). In addition, in a case-control study, the correlation between flavonoids from different dietary sources and the risk of CRC was evaluated, and it was found that specific flavonoids, especially flavonoids from vegetables and fruits, were negatively correlated with the risk of colorectal cancer (Xu et al., 2016). Other studies have shown that a high-fiber diet can increase the diversity of SCFA-producing bacteria and the expression of butyrate receptor in mice, thereby inhibiting colon cancer (Bishehsari et al., 2018).

### Fecal Microbiota Transplantation

In recent years, more and more studies have reported that fecal microbiota transplantation (FMT) has a good therapeutic effect on IBD, CRC, immune-related diseases, and other diseases. Routy et al. (Routy et al., 2018) found that intestinal flora improves the efficacy of anti-tumor immunotherapy based on PD-1, indicating that FMT can help fight tumors. The patients who responded to anti-PD-1 therapy had a varied species composition, such as *Bifidobacterium*, *E. faecalis*, etc. After anti-PD-1 treatment, the fecal bacteria from the patients with reaction can enhance the T cell effect and inhibit the development of tumor.

### Probiotics, Prebiotics, Synbiotics, and Postbiotics

At present, the use of probiotics in the treatment and prevention of cancer has become a research hotspot. Probiotics are living microorganisms that, when given in sufficient quantity, will bring health benefits to the host (Hill et al., 2014). The most common probiotic strains are *Bifidobacterium* and *Lactobacillus*. The study found that *Lactobacillus acidophilus* and *Lactobacillus plantarum* have a potential preventive effect in patients with polyps or CRC. The study used 16 S rRNA to conduct preliminary gene detection on 77 samples of CRC, polyps, and healthy subjects, and used absolute real-time PCR to determine the copy number of bacteria per Gram of feces. The results confirmed that taking *L. acidophilus* and *L. plantarum* in people with a family history of CRC and patients with polyps may be a method to prevent, treat, or alleviate CRC (Zinatizadeh et al., 2018). In addition, *Lactobacillus casei BL23*, a probiotic-specific strain, can inhibit colitis-associated CRC, which can be used as a potential new strategy for the prevention and treatment of CRC. In the CRC mouse model induced by azoxymethane/dextran sulfate sodium (AOM/DSS), *L. casei BL23* can reduce the histological score and proliferation parameters; reduce the level of cytokine IL-22; mediate the immunoregulation; upregulate the expression of caspase-7, caspase-9, and Bik; mediate the antiproliferative effect; and counteract the fecal flora imbalance induced by CRC in mice (Jacouton et al., 2017).

In addition, prebiotics are also a useful strategy to prevent CRC. Prebiotics are substrates that can be used by intestinal microorganisms and are beneficial to host health. The common prebiotics are fructans, inulin, fructooligosaccharides and galactooligosaccharides (Gibson et al., 2004; Bruno-Barcena and Azcarate-Peril, 2015). Galactooligosaccharides derived from lactulose (GOS-Lu) is a prebiotic preparation. In order to test its preventive effect on CRC, the *Rattus norvegicus* F344 animal model was established. The results showed that compared with the control group, the number of colon tumors in the GOS-Lu group was significantly reduced. At the same time, through the metagenomic sequencing of intestinal flora, it was found that the pro-inflammatory bacteria in the GOS-Lu group decreased significantly, while the beneficial bacteria increased significantly (Fernandez et al., 2018).

The combination of probiotics and prebiotics is called synbiotics, which have synergistic effects. Roller et al. found that the synbiotic combination of oligofructose-enriched inulin-based *Lactobacillus rhamnosus* and *Bifidobacterium lactis* can reduce the number of tumors by regulating the immune function in Peyer's patches, thus inhibiting the occurrence of CRC (Roller et al., 2004).

Postbiotics are soluble byproducts and metabolites secreted by intestinal microbiota, which have biological activity and thus interact with the host. Postbiotics have a protective effect on intestinal epithelial cells. Studies have shown that P40, a soluble protein derived from *L. rhamnosus GG*, can inhibit cytokine-induced epithelial cell apoptosis and intestinal barrier damage (Yan and Polk, 2012).

## Conclusions

CRC, as a disease induced by many factors, is closely related to diet, the environment, and lifestyle. Intestinal microbiota plays an important role in the development of CRC by destroying the homeostasis of the microenvironment, changing immune response, producing toxic metabolites, and directly or indirectly affecting epigenetic modification. Several types of bacteria have been proven to promote CRC in various ways and mechanisms. As a biomarker, intestinal microbiota provides a new method for the early diagnosis of noninvasive CRC. At the same time, intestinal flora in feces can also help predict CRC, thus reducing the occurrence and development of CRC. However, due to the complexity of carcinogenic mechanism, the best biomarker for disease diagnosis has not yet been determined. CRC can be managed through healthy diet, the use of probiotics, and fecal transplantation; however, each method has certain limitations, such as in the use of probiotics, there will be individual differences in probiotics, by which under different pathogenic conditions, its role will change accordingly, so the use of probiotics must be targeted. Intestinal microbiota can ferment dietary fiber to produce SCFAs. Some bacteria have been identified as potential butyric acid producers, including FN (Vital et al., 2014). Butyrate is one of the most produced SCFAs. It can pass through the intestinal epithelium and reach the lamina propria, directly forming the mucosal immune response (Tilg et al., 2018). Most studies have shown that butyrate can inhibit inflammation and canceration of the colon, but some studies have found contrasting results (butyrate paradox) in which the beneficial or harmful effects of butyrate on the mucosal immune system depend on its concentration and immune environment (De Almeida et al., 2019). In addition, dietary fiber intake is not enough to produce high levels of butyrate for the prevention of inflammation and cancer. Therefore, it is necessary to conduct more research on interventions involving high-level dietary fiber intake in the near future. With the deepening of various studies, the etiology and pathogenesis of CRC will be further revealed, and the role of microorganisms in the formation, progress, and therapeutic response of CRC will be further confirmed. Intestinal microorganisms is anticipated to become an important part of the prevention and treatment of CRC in the future.

## Abbreviations

AMPS, antimicrobial peptides; AOM, azoxymethane; BFT, *Bacteroides fragilis* toxin; CDT, cytolethal distending toxin; CRC, colorectal cancer; CutC, choline trimethylamine lyase; DSS, dextran sulfate sodium; ETBF, enterotoxigenic *Bacteroides fragilis*; IgA, immunoglobulin A; IBD, inflammatory bowel disease; IL-10 R A, IL-10 receptor alpha subunit mRNA; ROS, reactive oxygen species; SCFAs, short-chain fatty acids; SMO, spermine oxidase.
